# In situ laser fenestration of the Thoraflex Hybrid frozen elephant trunk for emergent revascularization of the left subclavian artery and laser fenestration for spinal cord perfusion

**DOI:** 10.1016/j.jvscit.2024.101426

**Published:** 2024-01-13

**Authors:** Maysam Shehab, Anders Wanhainen, Gustaf Tegler, Kevin Mani, Marek Kuzniar

**Affiliations:** a Division of Vascular Surgery, Department of Surgical Sciences, Uppsala University, Uppsala, Sweden; b Division of Surgery, Department of Surgical and Perioperative Sciences, Umeå University, Umeå, Sweden

**Keywords:** Hybrid FET, In situ laser fenestration, Left subclavian artery, Thoraflex

## Abstract

In situ laser fenestration (ISLF) has emerged as a promising technique for emergent revascularization of the left subclavian artery in the case of thoracic endovascular aortic repair coverage, presenting excellent technical success rates in most studies. We describe a case of ISLF of the Thoraflex Hybrid frozen elephant trunk device to achieve immediate left subclavian artery revascularization. We demonstrate the feasibility and technical success of using ISLF in this setting, providing a less invasive alternative to conventional surgical revascularization when required.

The left subclavian artery (LSA) might need to be covered in 26% to 40% of patients undergoing thoracic endovascular aortic repair (TEVAR) to achieve an adequate proximal seal.^1^ Revascularization of the LSA is recommended in elective TEVAR but can also be required in the acute setting. Various methods are available, such as carotid–subclavian bypass, subclavian artery transposition, and endovascular approaches using branched or fenestrated devices, as well as chimney techniques, all with pros and cons.^1^

In situ laser fenestration (ISLF) has emerged as a new promising technique for emergent revascularization of the LSA, presenting excellent technical success rates of >95% in most studies and an LSA patency rate of 97% to 100%.^2^ Previous studies have shown that Dacron-based stent grafts (eg, Zenith Alpha; Cook Medical Inc) are suitable for the ISLF technique. In contrast, polytetrafluoroethylene-based stent grafts (eg, Gore cTAG; W.L. Gore & Associates) should be avoided due to the potential liberation of toxic molecular substances after thermal ablation, the difficulty to perforate using a laser, and the propensity for fabric tears on dilatation of the fenestration.^2^^,^^3^

The Thoraflex Hybrid frozen elephant trunk (FET) device (Terumo Aortic) is a single-use medical device combining a Gelweave polyester graft with a nitinol self-expanding stent graft used for open surgical repair of the aortic arch and descending aorta.^4^ To the best of our knowledge, no studies have reported on ISLF of the Thoraflex Hybrid device. In this report, we describe a case of ISLF of a Thoraflex stent graft to achieve immediate LSA revascularization, demonstrating the feasibility and technical success of using ISLF in this setting. The patient provided written informed consent for the report of his case details and imaging studies.

## Case report

**Fig 1:**
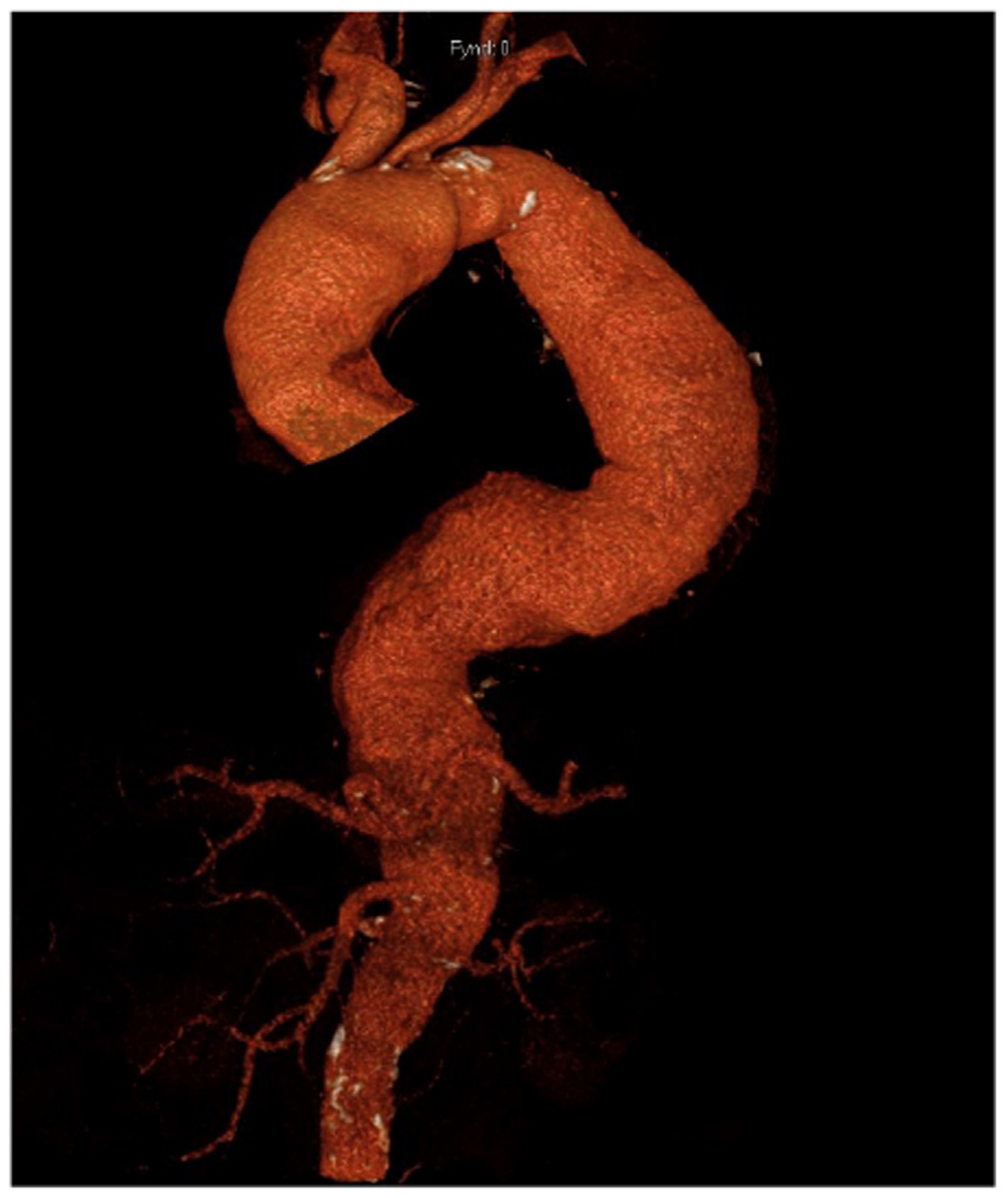
Three-dimensional reconstruction of preoperative computed tomography angiogram showing a Crawford type II aortic aneurysm.

A 75-year-old man presented with an 85-mm Crawford classification type II aortic aneurysm and a 57-mm ascending aortic aneurysm, with the largest diameter in the descending aortic aneurysm (Fig 1). A staged aneurysmal repair was planned to reduce the risk of paraplegia.

The first stage included ascending aortic repair with aortic valve replacement (23 mm; Magna Ease; Edwards Lifesciences) and FET, using a 28 × 30 × 180-mm Thoraflex Hybrid prosthesis. The distal anastomosis was performed at Ishimaru zone 2, with reimplantation of the brachiocephalic trunk and left carotid artery. The LSA was inaccessible and, therefore, left unreconstructed.

The immediate postoperative course was complicated by acute mesenteric ischemia, and the patient underwent thrombectomy of the superior mesenteric artery and stenting of the celiac artery with partial bowel resection. In addition, he developed a posterior cerebral infarction on the left side without residual symptoms. A follow-up computed tomography scan during the same admission depicted perfusion of the LSA from the nonsealed aneurysmal sac and a patent left vertebral artery.

Aneurysmal involvement of the visceral segment necessitated infrarenal sealing. The second stage was conducted 6 months later, with distal extension with TEVAR and four-fenestrated endovascular aortic repair, landing distally in the infrarenal aorta without a complete distal seal to temporarily sustain perfusion of the retrograde aneurysmal sac and LSA, reducing the risk of paraplegia. Our plan was to reconstruct the LSA with a bypass at the final stage of the procedure, together with distal prolongation for complete aneurysmal sac exclusion.

On postoperative day 1, the patient developed right arm motor weakness, sensory decline, ataxia, impaired cognition, and facial numbness. His blood pressure was significantly lower in the left than in the right arm (50 mm Hg systolic pressure difference). These symptoms resolved after increasing the mean arterial pressure to >90 mm Hg with noradrenaline infusion. A computed tomography scan showed no acute cerebral infarcts. However, no contrast flow was observed in the LSA during the arterial phase, and delayed venous phase imaging was not performed. No significant stenosis was identified in the extra- or intracranial internal carotid arteries or middle cerebral arteries, and both posterior communicating arteries were patent. A complementary duplex ultrasound scan showed low flow in the LSA (0.6-0.8 m/s) with reversed flow in the vertebral artery. The results of a neurologic consultation suggested cerebral hypoperfusion due to suspected subclavian steal syndrome.

**Fig 2:**
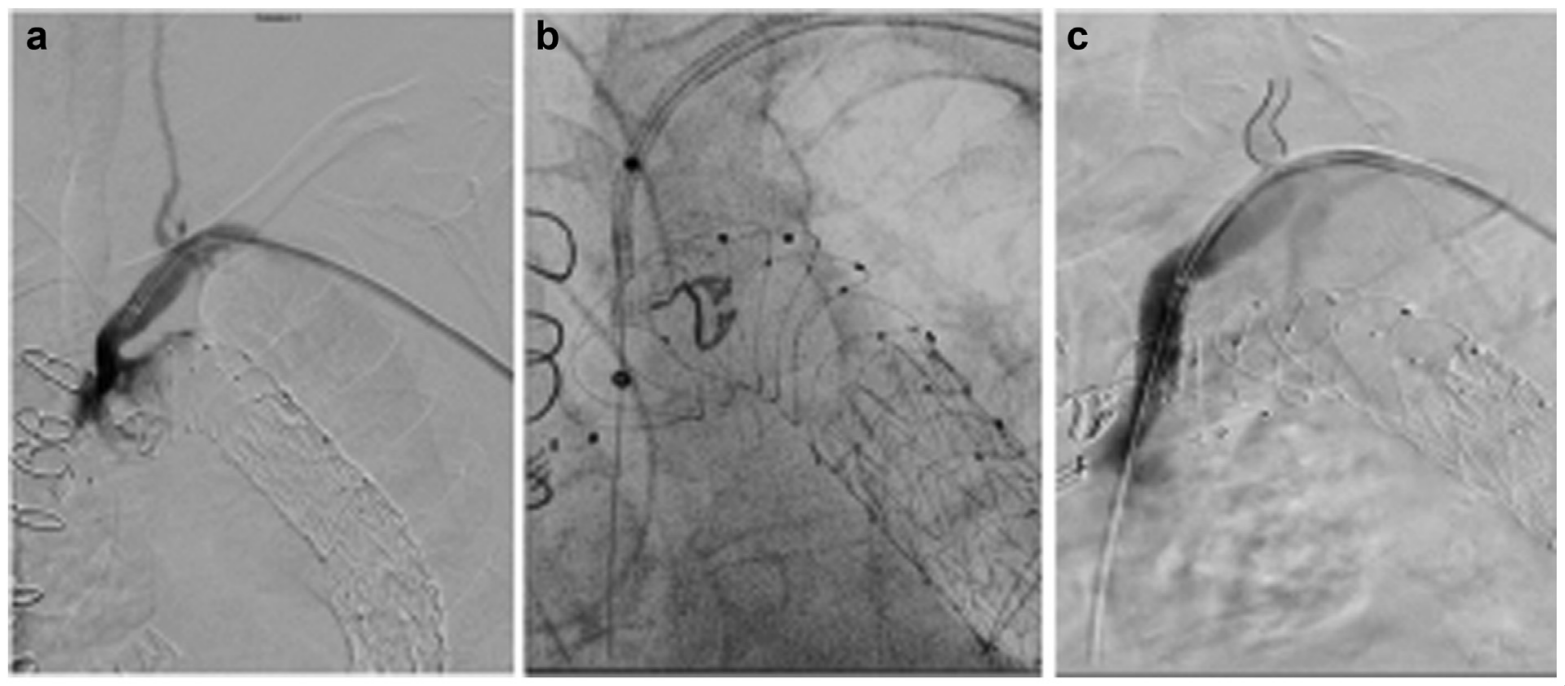
In situ laser fenestration (ISLF) of the Thoraflex stent graft. **a,** Diagnostic angiography showing flow in the left subclavian artery (LSA; *arrow*) and left vertebral artery (*dashed arrow*). **b,** The 0.018-in. wire was advanced into the Thoraflex and ascending aorta, and a 4 × 40-mm balloon (Armada 18; Abbott; *arrow*) was used to predilatate the fenestrated orifice. *Dashed arrow* indicates the Thoraflex stent graft. **c,** Completion angiogram showing a patent LSA and left vertebral artery.

To improve cerebral perfusion and lower the risk of paraplegia during the final-stage procedure, urgent retrograde ISLF to the LSA was performed. Under local anesthesia, exposure of the left brachial artery was performed. After administration of 5000 U of heparin, a TourGuide steerable sheath (8.5F diameter, 55 mm length; Medtronic Vascular) was advanced to the origin of the LSA and carefully adjusted to align perpendicularly to the Thoraflex stent graft. The alignment was verified in two different projections: a 50° left aortic oblique view and a barrel view. Retrograde fenestration of the Thoraflex stent graft was performed using a 308-nm CVX-300 excimer laser system (Spectranetics Corp, Philips Holding USA Inc) using a 2.3-mm, 0.018-in. laser catheter (Turbo Elite; Philips Healthcare). A 0.018-in. wire was advanced into the Thoraflex graft and ascending aorta, and a 4 × 40-mm, 0.018-in. balloon was used to predilatate the fenestrated orifice. The 0.018-in. wire was exchanged for a 0.035-in. Rosen wire (Cook Medical Inc), and the fenestration was further dilated with a 6 × 40-mm, 0.035-in. balloon, which was followed by deployment of a 10 × 38-mm balloon expandable covered stent (Advanta Atrium V12; Getinge). The distal part of the stent was additionally dilated with a 12 × 20-mm, 0.035-in. balloon to a nominal pressure to achieve a proper distal seal in the LSA. Completion angiography demonstrated a patent LSA stent, with no endoleak and antegrade filling of the left vertebral artery (Fig 2). The patient recovered from his neurologic symptoms at this time.

**Fig 3:**
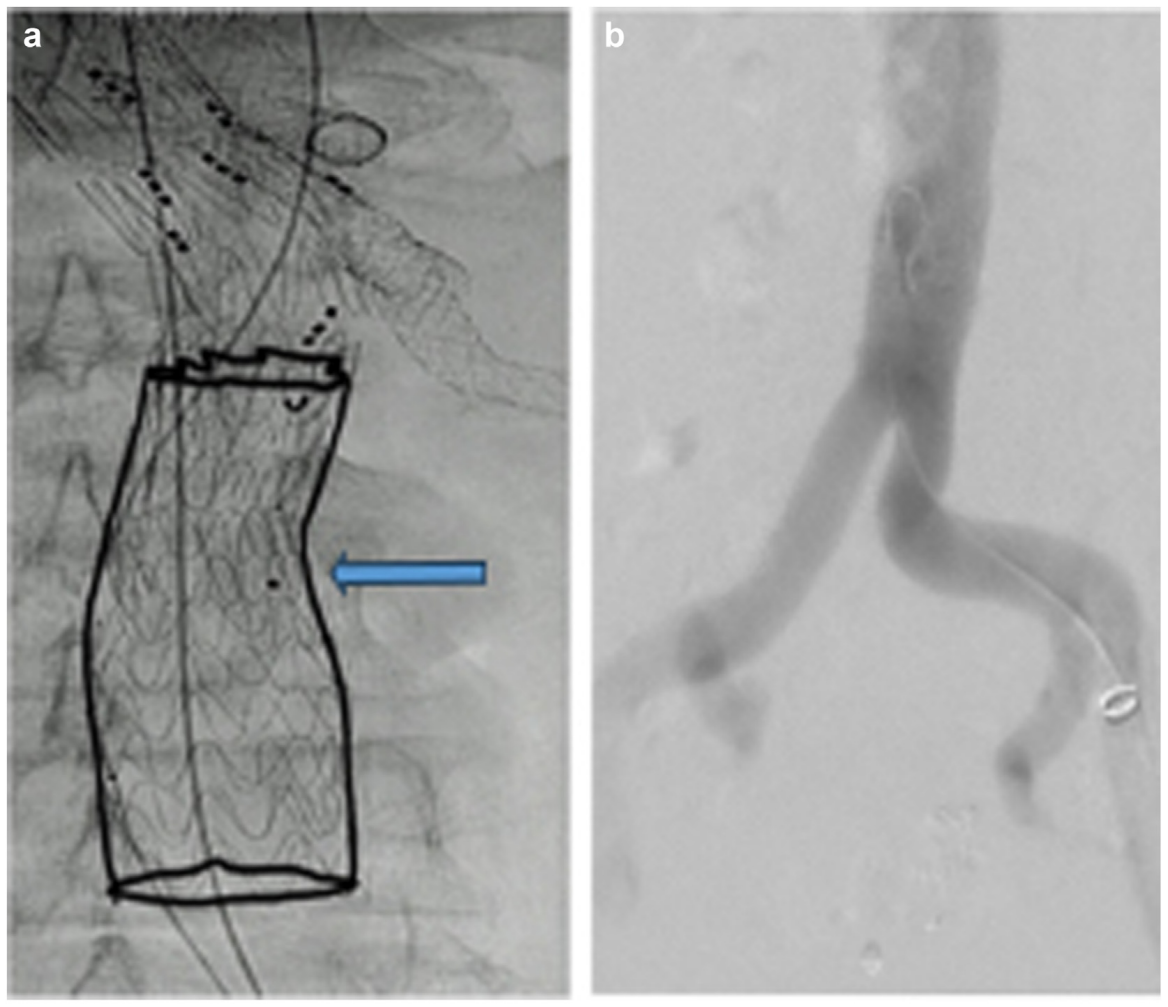
**a,** Distal extension with aortic tube graft and landing in the distal aorta (*arrow*). **b,** Completion angiogram showing no endoleak.

**Fig 4:**
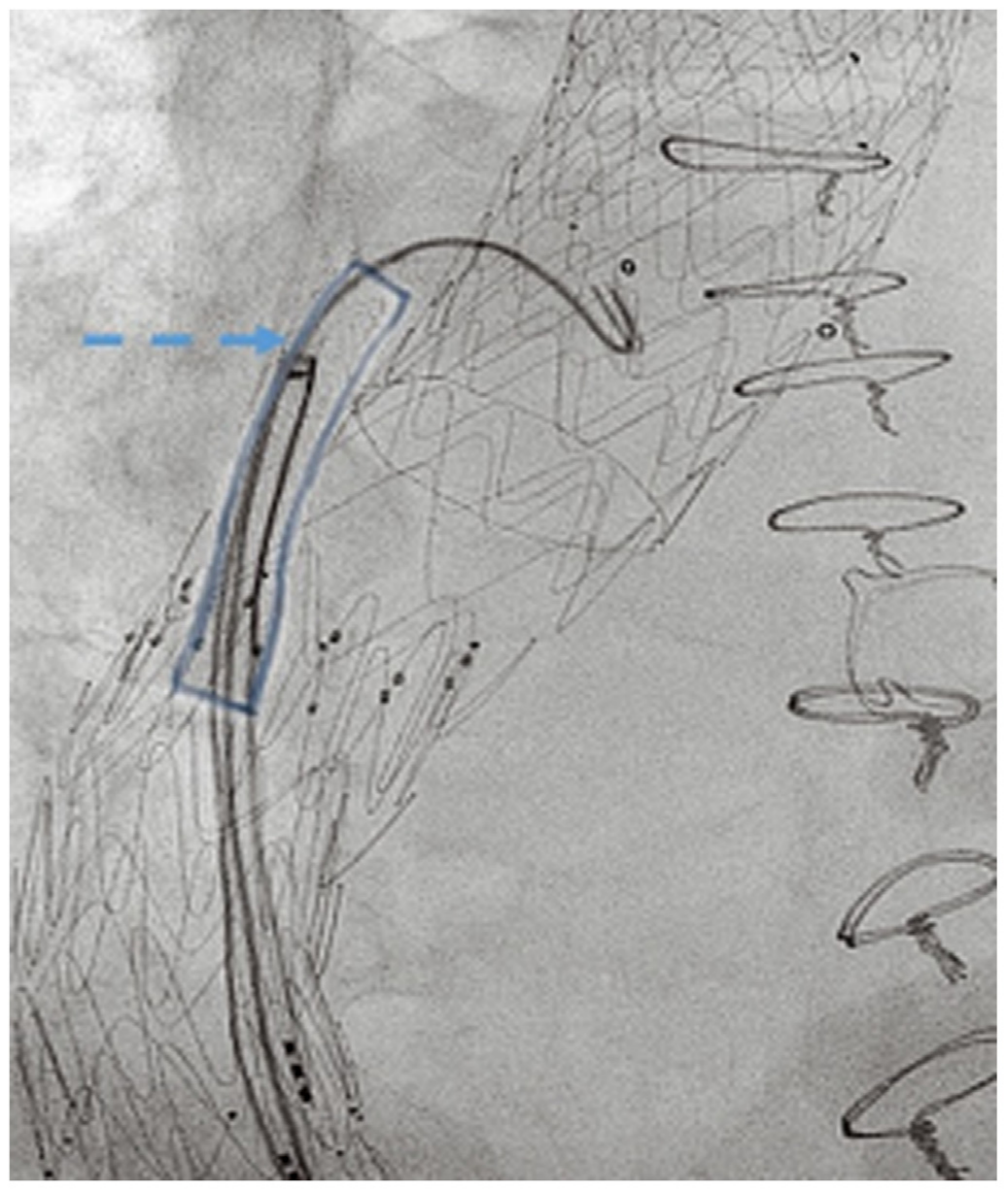
In situ laser fenestration (ISLF) to the thoracic stent graft was performed, and a stent was inserted for temporary aneurysm sac perfusion, highlighted in blue (*arrow*).

**Fig 5:**
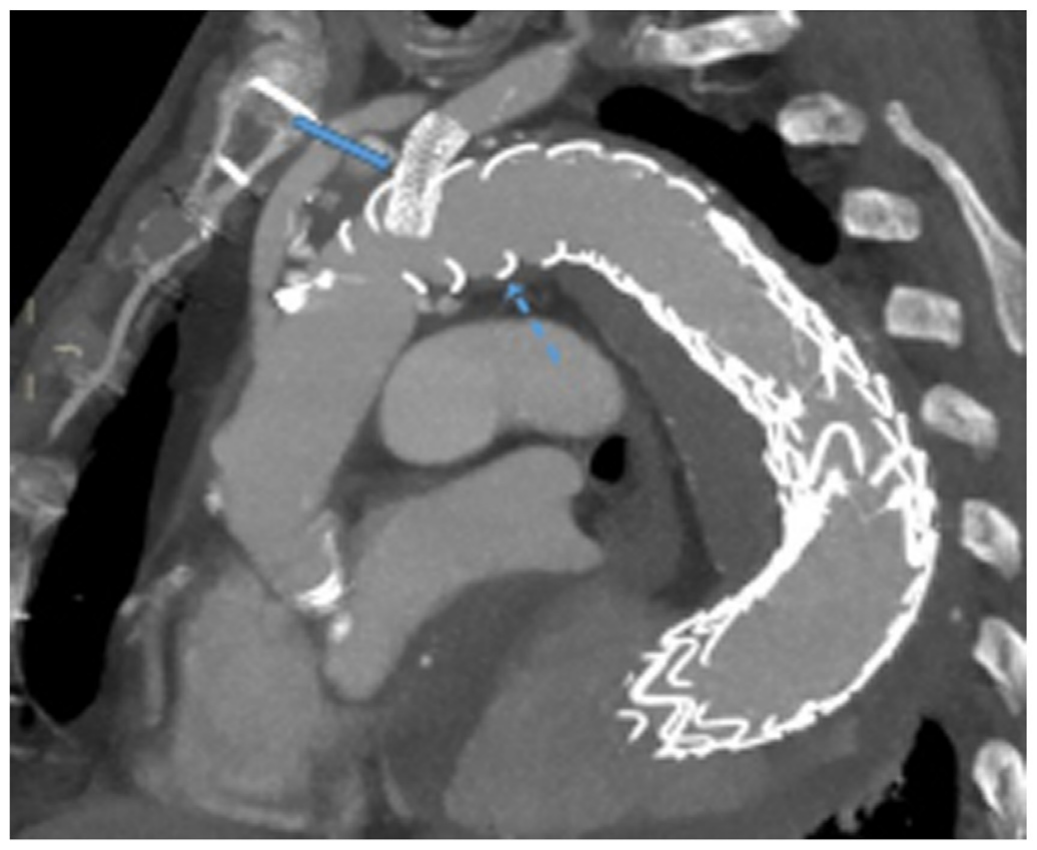
Three weeks after the last procedure, the patient had a follow-up computed tomography angiogram showing a patent left subclavian artery (LSA) stent (*arrow*) and no endoleak. *Dashed arrow* indicates the Thoraflex stent graft.

The third stage was performed 2 weeks later under local anesthesia, with distal extension using a Gore cTAG 28-28-100 mm stent graft (Gore Medical Inc), landing in the distal aorta with a satisfactory completion angiography (Fig 3). However, 12 hours later, the patient developed weakness in both legs (level B; American Spinal Injury Association scoring system). Spinal drainage was instituted immediately, along with blood pressure elevation, which resulted in only partial improvement. Therefore, an attempt was made to reperfuse the aneurysm sac through an ISLF of the distal thoracic stent graft (Fig 4). The patient was discharged home 10 days later with partial leg function. Three weeks later, a computed tomography angiogram demonstrated a patent LSA stent with no proximal endoleaks (Fig 5). Four weeks after discharge, he still had residual paraplegia. The distal fenestration was stented with a covered stent, and an Amplatzer vascular plug was placed 4 weeks later inside the stent to seal the aneurysm. The patient had no further neurological events.

## Discussion

For patients requiring extensive aortic and LSA coverage, LSA revascularization can be vital for spinal cord perfusion. Our initial plan to reconstruct the LSA during the FET procedure was altered due to inaccessibility. Relying on retrograde LSA perfusion and staging the procedure proved inadequate due to sac thrombosis at the LSA, indicating that earlier LSA revascularization would have been a better approach.

Extra-anatomic bypass remains the benchmark for LSA revascularization but can sometimes be unsuitable in the emergency setting and carries the risk of complications, such as thoracic duct injury, phrenic nerve injury, and stroke.^5^ Thus, interest has increased toward endovascular preservation of the LSA. Endovascular options include branched and fenestrated devices, which have limited off-the-shelf availability, especially in the European market.^6^ Parallel graft technology, such as snorkels or chimneys, is available in the emergency setting but is limited by its vulnerability to gutter leakage.^7^^,^^8^

ISLF represents a valuable off-the-shelf solution for aortic arch endovascular repair in the acute setting when a short window of opportunity exists for vascular reconstruction and for patients considered at high risk for conventional open management or who present with anatomic characteristics that do not permit application of custom-made solutions. ISLF technology, being energy-based, raises concerns of fabric fraying during balloon dilation, and limited data are available on the long-term durability and the frequency of endoleaks. Hence, ISLF is not primarily used in elective situations. Accumulating data, however, support its safety and feasibility with Dacron stent grafts in the short and medium term.^3^^,^^9^^,^^10^ A systematic review by Houérou et al^11^ indicated ISLF feasibility in treating aortic arch pathologies, with an incidence of type III endoleak of 3.5%, without further definition of whether these endoleaks were related to fabric tears or disconnection of the modules.

Although the focus of this case report was the feasibility of ISLF with the Thoraflex stent graft, the complex course of the present case also illustrates other applications of ISLF, such as creating a fenestration for temporary perfusion of the aneurysm sac. Kasprzak et al^12^ highlighted the potential of temporary aneurysm sac perfusion in maintaining spinal cord perfusion and possibly reducing spinal cord ischemia. Nevertheless, the level of evidence for its value remains weak, and the risk of aneurysm rupture must be considered. Given the high mortality and morbidity associated with paraparesis, and the elective nature of the repair, extending aneurysm sac perfusion for a few weeks was deemed a justified approach.

This case report demonstrates the feasibility of ISLF with the Thoraflex stent graft, which did not require any modifications compared with ISLF with other Dacron stent grafts. The case further shows the possibility of urgent innovative measures with the technology.

## Conclusions

This case report indicates that ISLF of the Thoraflex stent graft is technically feasible and can be considered as an alternative to standard surgical revascularization in the acute setting or for patients unfit for surgery. However, we cannot yet draw conclusions regarding the long-term durability of this nonreinforced fenestration.

## Disclosures

None.
